# Parental Migration for Work and Psychosocial Problems among Left-behind Adolescents in Nepal

**DOI:** 10.1007/s10903-025-01799-3

**Published:** 2025-10-28

**Authors:** Yagya Raj Adhikari, Edwin van Teijlingen, Pramod Raj Regmi, Sudip Khanal

**Affiliations:** 1 School of Health and Care, Faculty of Health, Environment and Medical Sciences, Bournemouth University, Bournemouth, United Kingdom; 2 Manmohan Memorial Institute of Health Sciences, Kathmandu, Nepal

**Keywords:** Migrant worker, Adolescence, Mental health, Well-being, South asia

## Abstract

Labour migration from Nepal has contributed significantly to the nation’s wealth, yet it has negatively affected the health and well-being of migrant workers. Until recently, limited research into the impact of parental migration on left-behind adolescents existed in Nepal. This study therefore aims to address this gap by conducting a cross-sectional study in Nawalparasi, one of the highest migrant-workers-sending districts in Nepal, involving 370 left-behind adolescents and 388 non-left-behind adolescents. Psychosocial well-being was measured using the Nepali version of Youth Paediatric Symptom Checklist (Y-PSC) and Strengths and Difficulties Questionnaire (SDQ). The survey was conducted in schools, and the overall response rate was 95.83 per cent. Most participants were in grade 10 (53.6%), with over half being female (51.2%) and a mean family size of 4.73 ± 1.52. Among them, 17 per cent experienced psychological problems, with left-behind adolescents showing a higher prevalence of low psychological well-being (20%) and psychological dysfunction (21%) compared to non-left-behind adolescents (13% and 14%, respectively). Adolescent females exhibited higher rates of psychological problems (21% vs. 12%) and dysfunctions (21% vs. 13%) than males in both groups. Perceived relationships with parents were strongly linked to psychosocial health, with left-behind adolescents being more likely to experience psychological issues and dysfunction compared to non-left-behind adolescents. Similarly, factors such as communication with migrated parents, visit intervals, social media use, and mobile access were associated with the psychosocial health of left-behind adolescents. The finding suggests that adolescents with migrant parents experienced poorer psychosocial well-being, suggesting that school-based mental health programs could help support their well-being.

**Supplementary Information:**

The online version contains supplementary material available at 10.1007/s10903-025-01799-3.

## Introduction

International migration for better employment opportunities is widespread in Nepal, and the trend is increasing [1]. Over five million Nepalese work abroad, mainly in Gulf Cooperation Council (GCC) countries, Malaysia and India [2]. Deep-rooted poverty and limited employment opportunities are the primary reasons for international labour migration from Nepal [3]. Whilst the increasing demand for labour from receiving countries, especially in the construction sector, is a major pull factor [4]. Despite the potential threat of work-related injuries and death [5], Nepalese migrants are taking risks associated with Gulf employment for better opportunities [6].

The Nepalese economy and household income depended highly on remittances, contributing about one-quarter of the country’s Gross Domestic Product (GDP) [7]. The Gulf countries alone contributed over 30 per cent of all remittances [8]. In addition, migrant workers’ earnings support households, particularly in rural areas, improving living standards, offering access to education and access, and creating small business investments [6].

As Nepal is one of the major countries supplying labour abroad, worker are separated from their families in different stages of their lives. While labour migration positively impacts GDP and household income [9, 10], studies have reported negative effects on the health and well-being of migrants themselves [11–14], as well as those left behind [15]. Research on the health and well-being of left-behind families in Nepal, such as wives of migrants and children, shows mixed outcomes [16–19]. However, there are limited studies about the effects on the health and well-being of left-behind adolescents whose parents work abroad.

Adolescence is a stage of life from ages 10 to 19 [20]. It is critical for physical, emotional, and psychological growth, and adolescents often need strong parental support [21, 22]. When parents are absent due to international migration, it can lead to mental health and well-being issues [23]. Thus, this study assessed the impact of parental work-related migration on the psychosocial health and well-being of left-behind adolescents whose parents migrated to GCC countries and Malaysia.

## Methods

### Study Design and Tools

This cross-sectional survey utilised a validated Nepali version of Youth Paediatric Symptom Checklist (Y-PSC) and Strength and Difficulties Questionnaire (SDQ). It was conducted in Nawalparasi district, which is one of the top ten districts for labour migrants receiving labour permits from 2008/09 to 2013/14 [24], and makes a significant contribution to the labour markets in the GCC and Malaysia [2].

### Study Population and Sampling

The population consisted of adolescents aged 14 to 19 recruited in grades 8 to 12 in selected schools. Participants are either left-behind adolescents whose parents (father, mother or both) migrated to the GCC countries or Malaysia for more than one year of employment, whilst non-left-behind adolescents are those who are staying with their parents in Nepal.

Three municipalities of the Nawalparasi Susta East with a high rate of outgoing migrants were identified by consulting with the district’s Migration Resource Centre (MRC) and a list of secondary schools was received from the District Education Office. Four schools were selected randomly from each municipality. From each school, students in the target age group were invited to participate in the survey. The calculated sample size for this study is 694 (347 for left-behind adolescents and 347 for non-left-behind adolescents). The sample size was calculated based on the prevalence rate of depression @ 11.7 per cent in a similar study in China [25].

The sample size was calculated based on the formula n = z^2^ P(1-P)/d^2^ [26] where:

z = z statistics for a level of confidence of 95% (i.e., 1.96).

P = prevalence of 1 (i.e., 0.05).

(10% non-response rate and design effect.

d = margin of error 5% in the proportion 2)

### Data Collection

Data collection was performed in June-July 2023. The survey package included a paper version of data collection tools and a participant information sheet (participant and parent/guardian versions) in Nepali. They were distributed in the classroom, allowing a week to decide whether to participate in the research. For participants under 18 years of age, our consent form required the signature of a parent or guardian. The researcher also visited the participants’ parental homes to provide assistance in reading the information sheet and to address any questions or concerns before giving their consent.

### Measurement Variables

Socio-demographic variables related to adolescents and their parents were collected and analysed with the outcome of the psychosocial well-being of participants. The Nepali version of the Youth Paediatric Symptom Checklist (Y-PSC) and Strengths and Difficulties Questionnaire (SDQ) tools were used to measure psychosocial well-being. Prior to going live with the study, the questionnaires were piloted with 35 adolescents to ensure that they are comprehensive, feasible and acceptable.

The Y-PSC–measures cognitive, emotional, and behavioural problems of adolescents [27]. The Y-PSC, used in this study, consists of 35 items rated as “Never,” “Sometimes,” or “Often,” with scores of 0, 1, and 2, respectively. The total score, ranging from 0 to 70, is the sum of individual item scores. The questionnaire was considered invalid if four or more items were missing, but one to three items missing were considered for analysis. A cut-off score of 30 was used to categorise the presence of psychosocial dysfunction [28].

The SDQ has 25 items comprising five sub-scales: emotional symptoms, conduct problems, hyperactivity/inattention, peer relationships problems and prosocial behaviour [29]. The first four sub-scales yield a total difficulties score of 0 to 40, and a higher total difficulties score indicates poorer psychological well-being. Each item is scored on a 3-point scale “not true” “somewhat true” and “certainly true” and values 0, 1 and 2, respectively. The threshold for psychological problems was taken as ≥ 17 based on studies conducted in Nepal [30].

### Data Management and Analysis

Data were entered into an Excel sheet, and the univariate, bivariate, and multivariate analyses were performed using R-Studio 2024.04.0. Bivariate analysis was used to examine the relationships between the dependent and independent variables, and p-values less than 0.05 were considered for further multivariate analysis. Multicollinearity was tested to perform the multivariate analysis. This study’s dependent variable was dichotomous, so a binary regression model was applied to calculate the odds ratio. The enter method in binary logistic regression helped identify significant variables at a 0.05 per cent and 95 per cent CI significance level.

### Ethics Consideration

Ethical Approval was sought from Bournemouth University (Ethics I.D. 44141) and Nepal Health Research Council (Reg. no. 274/2023). Written consent was taken from participants before data collection. In the case of minors (aged < 18), consent was taken from their parents/guardians. The questionnaire was free from any personal identifying details.

## Results

A total of 791 questionnaires were distributed, but 758 respondents completed the survey, with a response rate of 95.83%. Among them, 370 were left-behind adolescents, and 388 were non-left-behind adolescents.

The results show that participants studying in grade 10 were recorded as the highest (53.6%), followed by grade 11 (25.2%), grade 9 (16.6%) and grade 8 (4.6%). Over half of the respondents were female (51.2%), and the mean family member size was recorded as 4.73 ± 1.52. Most (49.2%) lived with their mother, and 42.9 per cent lived with their father and mother (both). However, only a small percentage of participants, 2.2 per cent, were reported living exclusively with their father (Table 1).

Most respondents (87.6%) perceived their relationship with parents as “very good”. Around half of them were Janajati (50.4%), 37.9 per cent were Brahmin/Chhetri/Thakuri followed by Dalit (11%), Madeshi (0.4%) and Muslim (0.3%). The majority of respondents was Hindu (93.9%), and the the religion of the remainder was Buddhism, Kirat, or Christianity. The mean age of the respondents was 15.99 ± 1.40 years, and the average number of dependent family members was 3.60 ± 1.40. Similarly, the mean duration of parental migration was 7.08 ± 5.29 years.

**Table 1:** Socio-demographic characteristics of respondents

Characteristics of the respondents	Category	Number	Per cent
Class	Grade 8	35	4.6
	Grade 9	126	16.6
	Grade 10	406	53.6
	Grade 11	191	25.2
Sex	Male	370	48.8
	Female	388	51.2
Family size	≤ 4	392	51.7
	> 4	366	48.3
	$$\:\stackrel{-}{x}\pm\:\sigma\:(4.73\hspace{0.17em}\pm\:\hspace{0.17em}1.52)$$		
Stay with	Mother	373	49.2
	Father	17	2.2
	Both	325	42.9
	Others	43	5.6
Perceived relationship with parents	Very Good	664	87.6
	Satisfactory	72	9.5
	Poor	19	2.5
	Very Poor	3	0.4
Caste/Ethnicity	Brahmin/Chhetri/Thakuri	287	37.9
	Madeshi	3	0.4
	Dalit	84	11
	Janajati	382	50.4
	Muslim	2	0.3
Religion	Hindu	712	93.9
	Buddhist	21	2.8
	Kirat	2	0.3
	Christian	23	3.0
Age (in Years)	≤ 14	115	15.2
	> 14	643	84.8
	$$\:\stackrel{-}{x}\pm\:\sigma\:\:(15.99\hspace{0.17em}\pm\:\hspace{0.17em}1.40)$$		
Number of family members dependent	< 3	177	23.4
	≥ 3	581	76.6
	$$\:\stackrel{-}{x}\pm\:\sigma\:\:(3.60\hspace{0.17em}\pm\:\hspace{0.17em}1.40)$$		
Parental migration (*n* = 370)	Father	338	91.4
	Mother	26	7.0
	Both	6	1.6
Duration of parental migration (years)	< 7	195	52.7
	≥ 7	175	47.3
	$\:\stackrel{-}{x}$ ±$\:\sigma\:$(7.08 ± 5.29)		

### Psychological Problems among Adolescents (SDQ)

**Fig. 1:**
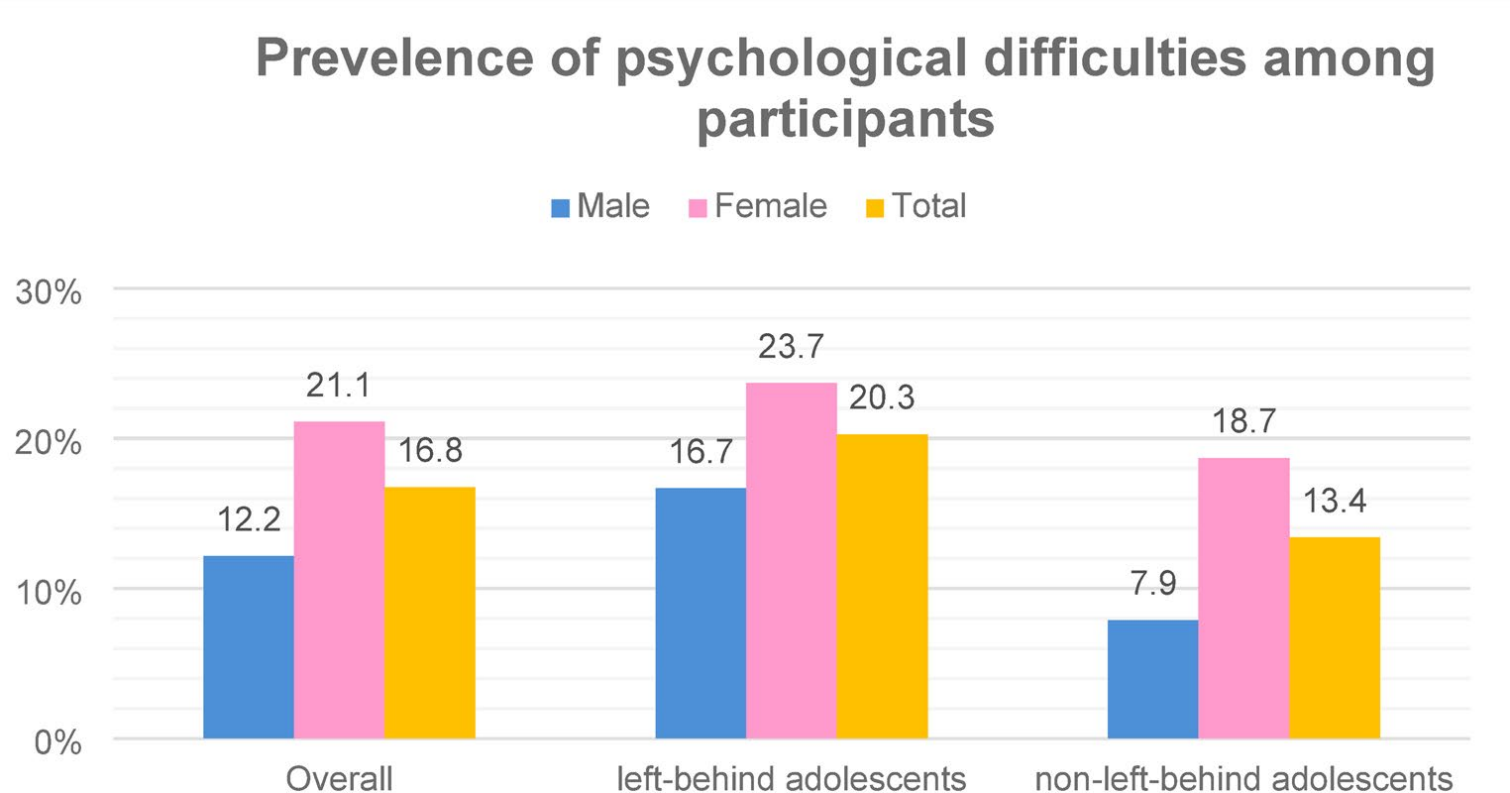
Psychological difficulties among adolescents

Figure 1 illustrates the prevalence of social psychological problems among respondents based on the Strengths and Difficulties Questionnaire (SDQ). Overall, 16.8 per cent of respondents experienced psychological problems. Female participants exhibited a higher prevalence of these issues (21.1%) than males (12.2%). Furthermore, a greater proportion (20.3%) of participants with low psychological well-being was observed in left-behind adolescents compared to non-left-behind adolescents (13.4%). Notably, both left-behind adolescents and non-left-behind adolescents showed higher rates of psychological problems among female participants, with 23.7 per cent of left-behind adolescents and 18.7 per cent of non-left-behind adolescents experiencing such issues.

### Psychosocial Dysfunction (Y-PSC)

**Fig. 2:**
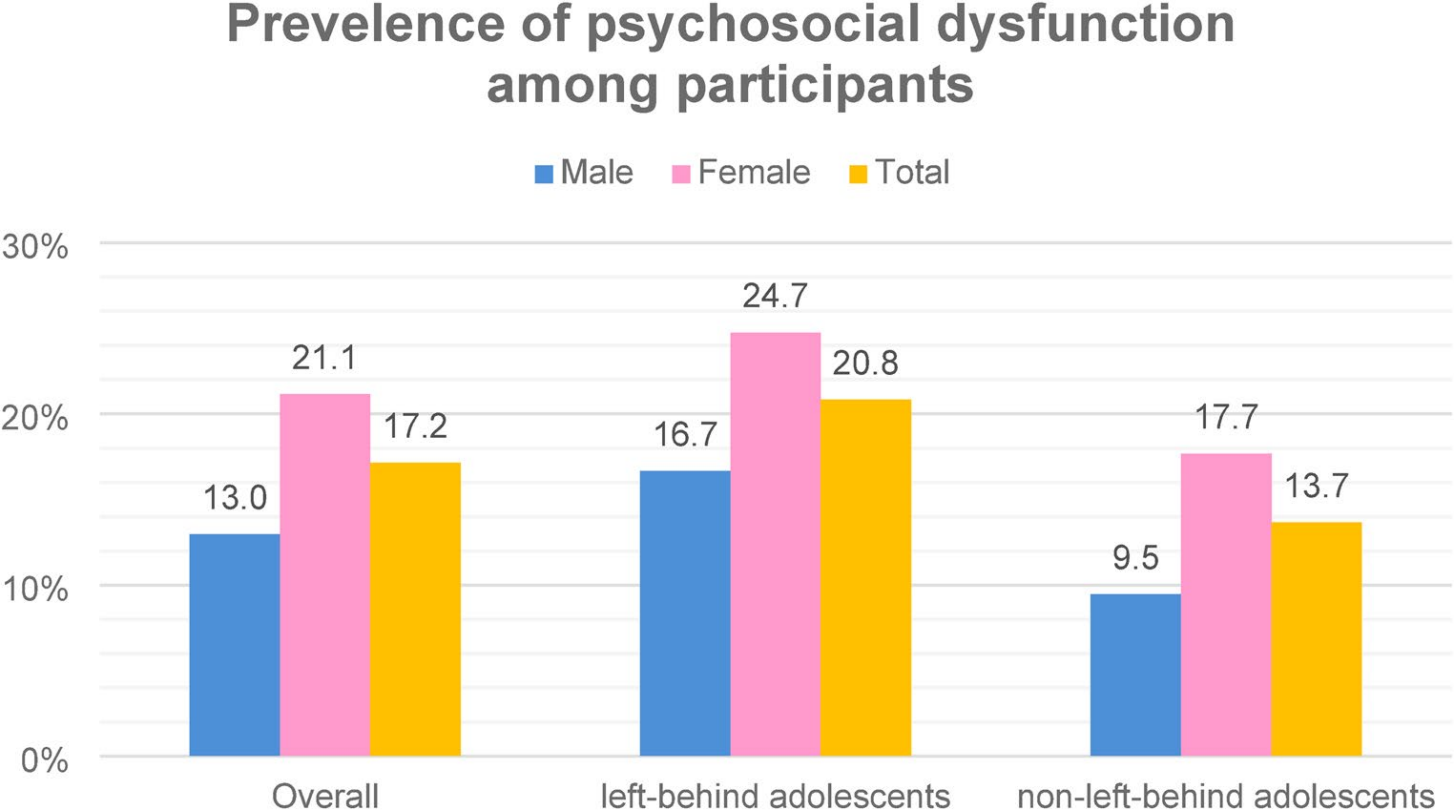
Psychosocial problems among participants

About one-sixth (17.2%) experienced psychosocial dysfunction, with fewer male respondents than females (13.0% vs. 21.1%) reporting such difficulties. Likewise, a higher rate of psychological dysfunction was observed in left-behind adolescents (20.8%) compared to non-left-behind adolescents (13.7%). Furthermore, within left-behind adolescents, female participants exhibited a higher rate (24.7%) of psychosocial dysfunction than males (20.8%) (Fig. 2).

### Factors Associated with Psychological well-being (SDQ)

Table 2 illustrates the multivariate logistic regression of the adolescents’ psychological well-being and various characteristics of the respondents. This indicates that female adolescents were 1.12 times more likely (AOR: 1.12, CI = 1.04–1.19) to have a social psychological problem than male adolescents of non-left-behind adolescents. The results show that perceived relationships with parents had a significant impact on the psychological well-being of the adolescents in both left-behind adolescents and non-left-behind adolescents.

In the non-left-behind adolescents group, the respondents who perceived the relationship with their parents as being (very) poor were 1.30 times more likely (OR: 1.30, CI = 1.06–1.59) to have psychological problems than respondents who have very good relationships with their parents among non-left-behind adolescents. Likewise, in the left-behind adolescents group, those with a satisfactory perceived relationship with their parents were 1.18 times (OR: 1.18, CI = 1.03–1.35) and poor and very poor was 1.41 times (OR: 1.41, CI = 1.10–1.18) more likely to be in social psychological problem compared to respondents whose perceived relationship with their parents was very good (Table 2).

In addition, this study showed that the frequency of parents returning from abroad to visit their family also significantly impacted the psychological well-being of the left-behind adolescents. Parents returning from abroad in two or three years are likely to have 1.17 times (OR: 1.17, CI = 1.05–1.30) psychological problems compared to those parents returning from abroad in one in two years.

Our study suggests that ways of communication and who most frequently initiates the call (adolescent or parents) significantly impacted the psychological well-being of left-behind adolescents. If the parents usually initiated the phone call primarily with their children, the adolescents were 1.15 times (OR: 1.15, CI = 1.04–1.27) more likely to have poor psychological problems as compared to those who communicate equally.

**Table 2:** Binary logistic regression between selected characteristics among left-behind adolescents and non-left-behind adolescents (SDQ)

Characteristics of Respondents	Category	Left-behind adolescents				Non-left-behind adolescents			
		COR (95% CI)	P-value	AOR (95% CI)	P-Value	COR (95% CI)	P-value	AOR (95% CI)	P-Value
Grade	8–9					Ref			
	10–11					1.08 (1.00–1.16.00.16)	0.037*	1.06 (0.98–1.14)	0.133
Sex	Male					Ref			
	Female					1.11 (1.04–1.19)	0.002*	1.12 (1.04–1.19)	0.004*
No of family member	≤ 4	Ref							
	> 4	1.10 (1.01–1.19)	0.026*	1.06(0.97–1.15)	0.180				
Perceived relationship	Very Good	Ref				Ref			
	Satisfactory	1.18 (1.03–1.36)	0.018*	1.18 (1.03–1.35)	0.021*	1.14 (1.01–1.28)	0.028*	1.11 (1.00–1.25.00.25)	0.061
	Poor/Very Poor	1.45 (1.14–1.84)	0.003*	1.41 (1.10–1.80)	0.007*	1.28 (1.05–1.57)	0.017*	1.30 (1.06–1.59)	0.011*
Personal mobile phone	No	1.11 (1.02–1.20)	0.017*	1.09 (1.01–1.18)	0.030*				
	Yes	Ref							
Television	No	1.10 (1.01–1.19)	0.035*	1.04 (0.95–1.19)	0.417				
	Yes	Ref							
Internet	No	1.13 (1.02–1.26)	0.020*	1.06 (0.95–1.19)	0.281				
	Yes	Ref							
Having social media	No					Ref			
	Yes					1.13 (1.01–1.26)	0.040*	1.10 (0.99–1.24)	0.085
Time to come from abroad	One Year	1.05 (0.93–1.18.93.18)	0.432	1.12 (0.99–1.25)	0.066				
	One - two years	Ref							
	Two - three years	1.17 (1.05–1.30)	0.005*	1.17 (1.05–1.30)	0.003*				
	Three - four years	1.02 (1.03–1.38)	0.735	1.04 (0.91–1.19)	0.570				
	Five years	1.20 (1.03–1.38)	0.016*	1.15 (0.99–1.33)	0.067				
Ways of communication	Phone	1.11 (0.99–1.25)	0.076	1.10 (0.98–1.24)	0.097				
	Mobile Texts	1.19 (1.00–1.41.00.41)	0.049*	1.10 (0.93–1.31)	0.271				
	Internet	Ref							
Mostly called by	Yourself	1.07 (0.90–1.28)	0.455	0.97 (0.82–1.16)	0.769				
	Parents	1.14 (1.03–1.26)	0.010*	1.15 (1.04–1.27)	0.006*				
	Both equally	Ref							

*Significant at 5% level of significance

COR: Crude odds ratio, AOR: Adjusted odds ratio, CI: Confidence Interval

### Factors Associated with Psychological well-being (Y-PSC)

Table 3 shows the multivariate logistic regression analysis between selected characteristics (or variables) and psychosocial dysfunction among adolescents, as measured by the Y-PSC. In the case of left-behind adolescents who perceived the relationship with their parents to be (very) poor were 1.39 times more likely to have psychological dysfunction (OR: 1.39, CI = 1.08–1.78) and those reporting satisfactory relations were 1.25 times more likely (OR: 1.25, CI = 1.09–1.44) than respondents who perceived the relationship with their parents to be very good.

Compared to left-behind adolescents who reported that their parents would return in one to two years, the left-behind adolescents who reported that their parents would return in two to three years were 1.14 times more likely (OR: 1.14, CI = 1.03–1.27) to have psychosocial dysfunction. Additionally, the left-behind adolescents who reported that parents mostly initiated phone calls were 1.17 times more likely (OR: 1.17, CI = 1.06–1.30) to have psychosocial dysfunction compared to left-behind adolescents who reported that calls were mostly made by both equally.

For non-left-behind adolescents, females were 1.09 times more likely (OR: 1.09, CI = 1.02–1.17) to have psychological dysfunction as compared to male left-behind adolescents. Moreover, non-left-behind adolescents who reported the perceived relationship with their parents as poor and very poor were 1.29 times more likely (OR: 1.29, CI = 1.05–1.58) to have psychological dysfunction than non-left-behind adolescents who reported perceiving a very good relationship with their parents. Furthermore, the result shows that non-left-behind adolescents who use social media were 1.12 times more likely (OR: 1.12, CI = 1.00–1.26.00.26) to have poor psychological dysfunction than non-left-behind adolescents (Table 3).

Further detailed analyses of the SDQ and the Y-PSC show the associations between psychological difficulties measured by SDQ and selected characteristics of respondents (Supplementary Table 1), and the associations between psychosocial dysfunction measured by the Y-PSC and selected characteristics of respondents (Supplementary Table 2) in each case comparing left-behind adolescents with non-left-behind adolescents.

**Table 3:** Binary logistic regression of selected characteristics of left-behind adolescents and non-left-behind adolescents (Y-PSC)

Characteristics of Respondent	Category	Left-behind adolescents				Non-left-behind adolescents			
		COR (95% CI)	P-value	AOR(95% CI)	P-Value	COR (95% CI)	P-value	AOR(95% CI)	P-Value
Sex	Male					Ref			
	Female					1.08 (1.01–1.16)	0.020*	1.09 (1.02–1.17)	0.012*
No of family members	≤ 4	Ref							
	> 4	1.11 (1.02–1.21)	0.031*	1.07 (0.98–1.166)	0.128				
Perceived relationship	Very Good	Ref				Ref			
	Satisfactory	1.25 (1.09–1.44)	0.002*	1.25 (1.09–1.44)	0.002*	1.13 (1.01–1.27)	0.034*	1.12 (0.99–1.25)	0.063
	Poor and Very Poor	1.45 (1.14–1.84)	0.003*	1.39 (1.08–1.78)	0.010*	1.28 (1.04–1.57)	0.019*	1.29 (1.05–1.58)	0.015*
Personal mobile phone	No	1.09 (1.01–1.19)	0.034*	1.08 (1.00–1.17.00.17)	0.058				
	Yes	Ref							
Television	No	1.10 (1.01–1.20)	0.031*	1.03 (0.94–1.12)	0.573				
	Yes	Ref							
Internet	No	1.17 (1.05–1.30)	0.004*	1.10 (0.98–1.23)	0.098				
	Yes	Ref							
Having social media	No					Ref			
	Yes					1.13 (1.01–1.27)	0.037*	1.12 (1.00–1.26.00.26)	0.046*
Time to come from abroad	One Year	1.01 (0.91–1.16)	0.634	1.11(0.98–1.24)	0.090				
	One in Two Year	Ref							
	Two or Three Year	1.14 (1.02–1.27)	0.022*	1.14 (1.03–1.27)	0.016*				
	Three or Four Year	1.01(0.88–1.16)	0.939	1.03 (0.90–1.18)	0.671				
	Five year	1.21(1.04–1.40)	0.014*	1.16 (1.00–1.34.00.34)	0.051				
Ways of communication	Through Phone	1.11(0.98–1.25)	0.089	1.10 (0.98–1.24)	0.113				
	Through Mobile Texts	1.23 (1.04–1.47)	0.016*	1.14 (0.96–1.36)	0.138				
	Internet	Ref							
Mostly called by	Yourself	1.07 (0.89–1.28)	0.458	0.97 (0.81–1.15)	0.710				
	Parents	1.17 (1.06–1.30)	0.002*	1.17 (1.06–1.30)	0.001*				
	Both equally	Ref							

*Significant at 5% level of significance

COR: Crude odds ratio, AOR: Adjusted odds ratio, CI: Confidence Interval

## Discussion

This is the first study to assess the psychological well-being of left-behind adolescents whose parents migrated to the Gulf or Malaysia to work. The study found that 16.8 per cent and 17.2 per cent of adolescents had poor mental well-being, as estimated by SDQ and Y-PSC, respectively. The rate of prevalence is in line with the previous studies conducted in the central part of Nepal [28] but higher than (14.2%, emotional and behavioural problems) the survey conducted in 16 districts of Nepal [31]. However, a community-based pilot study of the National Mental Health Survey in Nepal, conducted in three districts, reported that 11.2 per cent of adolescents (13 to 17 years) were suffering from mental disorders [32]. The variation could be due to the research utilising different tools and performed in a broader population. The World Health Organization (WHO) suggests that around 14 per cent of the global population aged 10 to 19 face some kind of mental health conditions [33]. Our result indicated a higher level of psychological problems in adolescents than globally, where many parents are migrating to GCCs and Malaysia for employment purposes.

We found that left-behind adolescents have poorer psychological well-being compared to non-left-behind adolescents. In contrast, a similar study in the far western region of Nepal [34] that employed the SDQ to assess the psychological problems of adolescents reported that non-left-behind adolescents were more likely to experience poor psychological well-being than left-behind adolescents. This discrepancy could be attributed to many parents of left-behind adolescents migrating to high-income countries like Japan, potentially providing their children with more financial security and a sense of pride. On the contrary, our study was conducted with adolescents whose parents worked in the GCCs or Malaysia, mostly in so-called unskilled, dirty, demanding or dangerous (3Ds) jobs [17]. A systematic review and meta-analysis [23] highlighted the negative impact on the mental health of left-behind adolescents and children, a result that aligns with the findings of this study. A school-based survey in Nepal [28] reported that adolescents living with single parents had a greater risk of poor psychological well-being compared to those living with both parents, which is in line with our study findings. The results of our study show that the majority of the non-left-behind adolescents are staying with single parents due to one of the parents migrating to the GCC or Malaysia for work.

Earlier studies in different countries showed mixed findings about the impact of parental international migration on adolescent psychological well-being. In Africa, no notable difference in mental well-being was observed between adolescents living with both parents and those with either their father or both parents residing abroad in Ghana and Nigeria [34]. However, in Angola, adolescents whose one or both parents lived abroad showed poorer psychological well-being than those who lived with both parents [35].

Our findings align with some of the studies conducted in China, where the social and cultural context differs significantly from our study location. These studies often reported poorer mental health outcomes [36–38] due to separation from parents. A recent study in Pakistan also revealed absence of migrant fathers may increase emotional and psychological distress among left-behind adolescents [39]. This absence manifests feelings of loneliness, abandonment, and emotional vulnerability, which can negatively influence their overall mental well-being. In our study, the left-behind category shows that most adolescents (91.4%) have fathers who migrated for work, which aligns with this result. Nepalese society is patriarchal in nature, where the father or eldest male makes important decisions, including those child-rearing and family matters [40]. Adolescents staying with their mother in rural Nepal may not feel as secure due to the absence of their father.

Our findings show that the perceived relationship with parents is one of the primary factors for psychosocial problems in left-behind adolescents, which was also reported by a large study (*N* = 1431) in the Philippines with students aged 11–17 years, which found that better parent-child relationships increased children’s emotional, psychological, and social well-being [41].

This study found that a higher percentage of female adolescents experienced psychosocial problems compared to male participants, although no significant gender differences were observed in the left-behind adolescents. However, a significant gender difference was found in the non-left-behind adolescents. In contrast, a school-based study conducted across 16 districts in Nepal with adolescents aged 11–18 reported no gender differences in emotional and behavioural problems [31]. Similarly, a recent study in Jordan with 8,000 participants (children/adolescents aged 8–18) reported a higher prevalence of mental health issues in females [42]. In Nepal, women are more vulnerable to mental health problems than men due to underlying societal and structural issues, which may contribute to gender disparities [43]. The study found that time intervals for visiting their home by migrant parents also significantly impact the psychological well-being of left-behind adolescents. This could be because those whose parents return home every one to two years feel more secure and experience a stronger emotional connection, which helps mitigate the feelings of abandonment and stress often associated with parental migration. Perhaps regular visits give these adolescents reassurance, stability, and a renewed sense of attachment, which can positively influence their mental and emotional health.

### Strengths & Weaknesses

This study is the first to measure the psychological well-being of adolescents whose parents worked abroad and compare them with similar adolescents whose parents stayed in Nepal. The psychological difficulties score from the SDQ and psychosocial dysfunction from Y-PSC complement each other. As mental health is not openly discussed in Nepali culture, the self-administered nature of our study made our participants more comfortable, thereby encouraging participants to provide honest and reliable data. Although the study sites were in parts of the country with a relatively large proportion of the population working abroad, it is not necessarily representative of the whole country. The survey was conducted in schools and did not include early school dropouts or those absent from school that day.

## Conclusion

This study examined the relationship between adolescents’ psychosocial well-being and parental migration to GCC countries and Malaysia for employment. Our findings show that adolescents with migrant parents experienced poorer psychosocial well-being compared to those whose parents do not migrate, but these differences are perhaps not as bad as one might have expected. In addition, the prevalence of psychosocial problems was higher in female adolescents than in males in both groups. Several factors were associated with the psychosocial well-being of left-behind adolescents, including the perceived relationship with parents, the frequency and nature of communication with migrated parents, and the intervals of visits home by the migrating parents. These elements significantly influence the emotional and psychological health of adolescents, as the physical absence of a parent can lead to feelings of insecurity or emotional distress. Furthermore, inconsistent or limited communication can exacerbate these challenges, making it more difficult for adolescents to maintain a strong emotional bond with their migrating parents. There is a potential growing social problem in countries with high numbers of their population working abroad whilst leaving their children behind.

### Recommendations

- We suggest further qualitative research is needed to gain insight into ways to improve relationships between left-behind adolescents and their parents working abroad. This could include interviews with the triad of the adolescent, their parent(s) abroad and their remaining parent/guardian in Nepal. In addition, a longitudinal study on left-behind adolescents may provide us with more robust evidence on psychosocial well-being and the risk factors. Also, we suggest further research from a gender perspective, as our findings indicate that female participants are more vulnerable to psychosocial health issues compared to males.
- Psychosocial health problems are found at alarming levels among both left-behind adolescents and non-left-behind adolescents, with a higher prevalence in left-behind adolescents. Therefore, school-based mental health promotion programs could be beneficial in addressing the psychosocial well-being of adolescents in rural Nepal. Similarly, it may be beneficial to raise awareness among migrant workers about the health impacts of migration on left-behind children. This can be done by including such topics in the mandatory pre-departure training for aspiring migrants.

## Supplementary Information

Below is the link to the electronic supplementary material.

Supplementary Material 1

## Data Availability

No datasets were generated or analysed during the current study.
