# Supplementary material for: Parental Migration for Work and Psychosocial Problems among Left-behind Adolescents in Nepal
Source: J Immigr Minor Health. 2025 Oct 28;28(2):429–38. doi: 10.1007/s10903-025-01799-3 (PMC13083506; doi:10.1007/s10903-025-01799-3)
Supplement: Supplementary file 1 — Supplementary Material 1 [file 10903_2025_1799_MOESM1_ESM.docx]

Supplementary Table 1. Association between psychological difficulties measured by SDQ and selected characteristics of respondents.

| Characteristics of respondents | Category | Psychological difficulties (left-behind adolescents) | | P-value | Psychological difficulties (non-left-behind adolescents) | | P-value |
| --- | --- | --- | --- | --- | --- | --- | --- |
|  |  | No (%) | Yes (%) |  | No (%) | Yes (%) |  |
| Grade | Grade 8 - 9 | 245 (81.1) | 57 (18.9) | 0.159 | 236 (89.1) | 29 (10.9) | 0.037* |
|  | Grade 10 - 11 | 50 (73.5) | 18 (26.5) |  | 100 (81.3) | 23(18.7) |  |
| Sex | Male | 150 (83.3) | 30 (16.7) | 0.093 | 175 (92.1) | 15 (7.9) | 0.003* |
|  | Female | 145 (76.3) | 45 (23.7) |  | 161 (81.3) | 37 (18.7) |  |
| No of family members | ≤ 4 | 172 (83.9) | 33 (16.1) | 0.026* | 163 (87.2) | 24 (12.8) | 0.752 |
|  | > 4 | 123 (74.5) | 42 (25.5) |  | 173 (86.1) | 28 (13.9) |  |
| Perceived Relationship | Very Good | 267 (82.4) | 57 (17.6) | 0.001* | 301 (88.5) | 39 (11.5) | 0.007* |
|  | Satisfactory | 23 (65.7) | 12 (34.3) |  | 28 (75.7) | 9 (24.3) |  |
|  | Poor & very poor | 5 (45.5) | 6 (54.5) |  | 7 (63.6) | 4 (36.4) |  |
| Personal Mobile Phone | No | 124 (74.3) | 43 (25.7) | 0.017* | 190 (88.4) | 25 (11.6) | 0.253 |
|  | Yes | 171 (84.2) | 32 (15.8) |  | 146 (84.4) | 27 (15.6) |  |
| Television | No | 99 (73.9) | 35 (26.1) | 0.035* | 116 (85.3) | 20 (14.7) | 0.577 |
|  | Yes | 196 (83.1) | 40 (16.9) |  | 220 (87.3) | 32(12.7) |  |
| Internet | No | 48 (69.6) | 21 (30.4) | 0.020* | 96 (85.0) | 17(15.0) | 0.543 |
|  | Yes | 247 (82.1) | 54 (17.9) |  | 240 (87.3) | 35(12.7) |  |
| Having social media | No | 39 (84.8) | 7 (15.2) | 0.362 | 37 (97.4) | 1(2.6) | 0.049* |
|  | Yes | 250 (78.6) | 68 (21.4) |  | 219 (81.1) | 51(18.9) |  |
| Time to come from abroad | One year | 61 (82.4) | 13 (17.6) | 0.020* |  |  |  |
|  | One – two years | 95 (87.2) | 14 (12.8) |  |  |  |  |
|  | Two – three years | 73 (71.6) | 29 (28.4) |  |  |  |  |
|  | Three – four years | 39 (84.8) | 7 (15.2) |  |  |  |  |
|  | Five years | 27 (69.2) | 12 (30.8) |  |  |  |  |
| Ways of Communication | Phone | 38 (71.7) | 15 (28.3) | 0.042* |  |  |  |
|  | Mobile texts | 15 (65.2) | 8 (34.8) |  |  |  |  |
|  | Internet | 242 (82.3) | 52 (17.7) |  |  |  |  |
| Mostly Call By | Yourself | 16 (76.2) | 5 (23.8) | 0.032* |  |  |  |
|  | Parents | 55 (69.6) | 24 (30.4) |  |  |  |  |
|  | Both equally | 224 (83.0) | 46 (17.0) |  |  |  |  |

**Significant at 5% level of significance*

Supplementary Table 2. Association between psychosocial dysfunction measured by the Y-PSC and selected characteristics of respondents.

| Characteristics of respondents | Category | Psychosocial dysfunction (left-behind adolescents) | | P-value | Psychosocial dysfunction (non-left-behind adolescents) | | P-value |
| --- | --- | --- | --- | --- | --- | --- | --- |
|  |  | No (%) | Yes (%) |  | No (%) | Yes (%) |  |
| Sex | Male | 150 (83.3) | 30 (16.7) | 0.056 | 171 (90.5) | 18 (9.5) | 0.020* |
|  | Female | 143 (75.3) | 47 (24.7) |  | 163 (82.3) | 35 (17.7) |  |
| No of family members | ≤ 4 | 172 (83.9) | 33 (16.1) | 0.018* | 163 (87.2) | 24 (12.8) | 0.634 |
|  | > 4 | 121 (73.3) | 44 (26.7) |  | 171 (85.5) | 29 (14.5) |  |
| Perceived Relationship | Very Good | 267 (82.4) | 57 (17.6) | <0.001*** | 299 (88.2) | 40 (11.8) | 0.009* |
|  | Satisfactory | 21 (60) | 14 (40) |  | 28 (75.7) | 9 (24.3) |  |
|  | Poor & Very Poor | 5 (45.5) | 6 (54.5) |  | 7 (63.6) | 4 (36.4) |  |
| Personal Mobile Phone | No | 124 (74.3) | 43 (25.7) | 0.046* | 189 (88.3) | 25 (11.7) | 0.258 |
|  | Yes | 169 (83.3) | 34 (16.7) |  | 145 (83.8) | 28 (16.2) |  |
| Internet | No | 46 (66.7) | 23 (33.3) | 0.004** | 94 (83.2) | 19 (16.8) | 0.325 |
|  | Yes | 247 (82.1) | 54 (17.9) |  | 240 (87.6) | 34 (12.4) |  |
| Having social media | No | 39 (84.8) | 7 (15.2) | 0.421 | 37 (97.4) | 1 (2.6) | 0.037* |
|  | Yes | 254 (78.4) | 70 (21.6) |  | 297 (85.1) | 52 (14.9) |  |
| Time to come from abroad | One year | 61 (82.4) | 13 (17.6) | 0.036* |  |  |  |
|  | One – two years | 93 (85.3) | 16 (14.7) |  |  |  |  |
|  | Two – three years | 74 (72.5) | 28 (27.5) |  |  |  |  |
|  | Three – four years | 39 (84.8) | 7 (15.2) |  |  |  |  |
|  | Five years | 26 (66.7) | 13 (33.3) |  |  |  |  |
| Mostly called by | Yourself | 16 (76.2) | 5 (23.8) | 0.009** |  |  |  |
|  | Parents | 53 (67.1) | 26 (32.9) |  |  |  |  |
|  | Both equally | 224 (83.0) | 46 (17.0) |  |  |  |  |
